# Exploring psychological experiences of soldiers who have undergone voluntary male medical circumcision at the Engineers Battalion in Kasungu, Malawi

**DOI:** 10.4102/hsag.v31i0.3086

**Published:** 2026-03-17

**Authors:** George Chapweteka, Nixon Chidzere, Thandie Munthali, Patson Kumwenda, Chimwemwe Munthali, Esmie Mkwinda, Geldine Chironda

**Affiliations:** 1 Department of Clinical Medicine, Faculty of Health Sciences, Saint John of God University, Mzuzu, Malawi; 2 Department of Nursing and Midwifery, Faculty of Health Sciences, Saint John of God University, Mzuzu, Malawi

**Keywords:** psychological experiences, soldiers, voluntary, male medical circumcision, Malawi

## Abstract

**Background:**

Voluntary medical male circumcision (VMMC) has gained recognition for its potential to reduce the risk of HIV transmission. However, limited academic research has comprehensively examined the psychological experiences of individuals who have undergone this procedure.

**Aim:**

The study aimed to explore the psychological experiences of soldiers who have undergone VMMC at the Engineers Battalion in Kasungu, Malawi.

**Setting:**

The study was conducted at the Engineers Battalion in Kasungu, Central Region of Malawi.

**Methods:**

A qualitative phenomenological study design was used. A purposive sampling strategy was used to enrol eight participants. A semi-structured interview schedule with probing questions was used to collect data. An interpretive thematic framework approach was used to analyse data.

**Results:**

Immediate psychological experiences included heightened anxiety, vulnerability and physical discomfort. Short-term experiences involved challenges adapting to physical restrictions and feelings of self-consciousness. Long-term psychological experiences were characterised by a blend of positive self-perceptions.

**Conclusion:**

The study highlights the mental health challenges associated with routine VMMC among an under-researched population in Malawi. Voluntary medical male circumcision significantly affects the psychological well-being of soldiers across various stages of recovery, underscoring the need to integrate psychological counselling and support into military health initiatives.

**Contribution:**

Findings from this study will contribute to the growing body of knowledge on the intersection between VMMC, psychological well-being and military health policies, ultimately informing the design of tailored interventions to support circumcised soldiers in Malawi and beyond.

## Introduction

Voluntary male medical circumcision (VMMC) is a surgical procedure involving the removal of the foreskin from the penis, typically conducted by trained healthcare professionals within clinical settings (Zhang & Vermund 2022). A study by Maheu-Giroux et al. (2021) reported that the global prevalence of VMMC among men aged 15–49 years increased from 10.4% in 2005 to 21.8% in 2015, reflecting substantial progress in scaling up VMMC services. Over the past years, VMMC has gained significant recognition in the global health sector as an effective strategy for reducing the transmission of HIV and other sexually transmitted infections (STIs) (Maibvise et al. 2024). In 2020, the World Health Organization (WHO) updated its guidelines to maximise the HIV prevention impact of safe VMMC services, emphasising the integration of these services into broader health initiatives targeting adolescent boys and men. The Malawi Demographic and Health Survey (MDHS) of 2020 indicated that approximately 41% of men aged 15–49 years in Malawi had undergone VMMC, reflecting the successful integration of VMMC into the national HIV prevention strategy (Mphepo et al. 2023; National Statistical Office [Malawi] & ICF 2017).

Voluntary Medical Male Circumcision reduces HIV transmission by 58% – 60% (Lancet Global Health 2023). These findings reinforce the role of VMMC as a critical component in comprehensive HIV prevention programmes. The WHO in 2021 further corroborated these findings, indicating a 60% reduction in HIV acquisition among circumcised men. As a result, VMMC is increasingly recognised as a crucial public health intervention (Weiss et al. 2024). Soldiers who perceived the procedure positively often cited improved health and cleanliness, similar to the positive long-term outcomes on sexual satisfaction. Voluntary Medical Male Circumcision programmes have been widely implemented in regions with high HIV prevalence, including sub-Saharan Africa (Luseno, Rennie & Gilbertson 2023; Bansi-Matharu et al. 2023), and have reached diverse populations, including soldiers and civilians.

Beyond its HIV preventive benefits, VMMC has also been acknowledged for its potential to reduce the transmission of other STIs, including syphilis and human papillomavirus (HPV) (Morris et al. 2019). Military personnel often operate in high-risk environments where the prevalence of STIs, including HIV, is heightened because of various socio-behavioural factors (Lasu et al. 2023). While the primary objective of VMMC is to bolster public health outcomes through HIV prevention, recent studies have increasingly turned their attention to the psychological experiences associated with this procedure (Nanteza et al. 2020). Some studies have explored the psychological aspects of VMMC, highlighting both positive and negative aspects of men’s experiences. For instance, research indicates that VMMC may lead to improved self-esteem and reduced anxiety concerning HIV risk (Herman-Roloff et al. 2011). Nonetheless, it is vital to recognise that individual psychological responses may vary, with some men experiencing concerns related to body image, sexual function or cultural perceptions.

While VMMC has gained recognition for its potential to reduce the risk of HIV transmission (Maibvise et al. 2024), limited academic research has focused on comprehensively examining the psychological experiences of individuals who have undergone this procedure. Thus, understanding the psychological dimensions of VMMC among these individuals remains an understudied area. This study aims to explore the psychological experiences of soldiers who have voluntarily undergone VMMC at the Engineers Battalion in Kasungu, Malawi.

## Research methodology

### Study design and setting

A qualitative, phenomenological design was used for the study, and in-depth interviews were used to collect data (Villa et al. 2018). The study was conducted at the Engineers Battalion in Kasungu in the Central Region of Malawi. This battalion is one of the major units in the country and is situated in the southern part of the district, 15 km from Kasungu town along the M1 road, and 3 km west of the M1 road. The battalion, which opened in 2012, comprises soldiers specialised in different fields, such as civil engineering.

### Population, sample and sampling

The total population of soldiers at the Engineers Battalion was 850, irrespective of being circumcised or not. The sample size of eight participants was determined by data saturation, which was the stage at which additional interviews were no longer yielding new information (Mwita 2022; Saunders et al. 2018). Purposive sampling was used, meaning participants were selected from those who had undergone the procedure within the previous year and were able to provide consent. The researcher purposively selected these participants because they possessed rich information on issues surrounding circumcision.

### Data collection tools

A semi-structured interview guide, developed through an in-depth literature review, was used (Creswell & Creswell 2017). The method of data collection was face-to-face. During interviews, probing and clarification techniques were employed to encourage participants to provide detailed descriptions. Follow-up questions were asked to obtain further elaboration. Audio recording equipment was used to capture the participants’ responses accurately and in their own words. A register was used to capture the names of soldiers who were circumcised, and a room was used during interviews. A minimum of 60 min was required for each participant. Participants who declined audio recording were excluded from the study.

### Data collection procedure

The data collection process occurred in November 2023. Participants were recruited from the Engineers Battalion. The clearance certificate from the Mzuzu Research Ethics Committee was used to negotiate permission to conduct the study at the Engineers Battalion, and the approval letter was presented as support for this request. A register was used to identify participants. Data were collected in English, as all the participants had at least attained the Malawi School Certificate of Education (MSCE) and were able to understand and comprehend the information without the need for translation into Chichewa. On the day of data collection, each participant was invited to the interview room. The purpose of the study was explained, and everyone was given an opportunity to ask questions or seek clarifications about the study and their participation. This was followed by the administration of the socio-demographic questionnaire and the interview guide, which included probes and follow-up questions.

For immediate psychological experiences (within 24 h after the procedure), participants were asked to describe their initial experiences and feelings following circumcision, including any discomfort, pain, concerns or complications during the immediate post-circumcision period. For the short-term psychological experiences (from 24 h to 7 days), participants were asked to describe the healing process following circumcision and identify any challenges or discomforts faced during this period. For the long-term psychological experiences (8 days to 1 year), participants were asked to describe how they adapted to the longer-term effects of circumcision, how their perceptions or feelings about the procedure changed over time, and whether any negative effects persisted in the long term.

### Trustworthiness of qualitative data

Trustworthiness in qualitative research ensures the rigour and credibility of a study’s findings, encompassing credibility, dependability, confirmability and transferability (Shenton 2004). Credibility was ensured through prolonged engagement, as the researcher spent time with the participants during data collection. Additionally, credibility was strengthened through frequent debriefing sessions between the researcher and the supervisor on the data collected. Moreover, peer debriefing, through discussions of the results with other academic peers, was conducted. Negative case analysis was enhanced by incorporating literature that contrasted the study’s findings in the discussion section. A detailed research process and the methods used to collect data were provided to enhance dependability.

An audit trail, describing the research steps taken from the start of the study to the reporting of findings, was maintained, and a record of the research history was kept by the researcher. Regarding confirmability, the interpretations and findings were derived directly from the data, with detailed conclusions and justifications. The researcher was careful not to allow prior knowledge of the area to cloud their judgment during data collection, analysis and report writing. Finally, transferability was addressed by providing a detailed, context-rich description of the study setting, allowing future researchers to assess the applicability of the findings to different contexts. These measures collectively enhanced the trustworthiness of the study, ensuring its quality and reliability.

### Data analysis

To achieve trustworthy and insightful findings, thematic analysis (Braun & Clarke 2020) was used to analyse the collected qualitative data. Data were transcribed from the audio recordings and organised according to the specific objectives of the study. The researcher familiarised herself with the data by listening to the audio and reading and re-reading the transcripts. Thereafter, the researcher began identifying meaningful texts with similar characteristics, thus developing initial codes (Braun & Clarke 2020). The researcher identified the code name and indicated the portion of the text associated with it. Codes were reviewed and sorted, and those that exhibited similarities were clustered to reflect a coherent and meaningful pattern in the data. A set of themes matching the coded data was then developed. After the initial development of themes, the researcher refined the set of themes derived from the coded data (Braun & Clarke 2020). Any inadequacies within the coded data were addressed, and themes were revised accordingly. The refined themes were defined and named to give the reader a clear sense of their meaning.

### Ethical considerations

An application for full ethical approval was made to the Mzuzu University and the Faculty of Health Science Research Committee and ethics consent was received on 30 October 2024. The ethics approval number is FOHS/STJOG/24/001. Thereafter, site approval was sought to interview the soldiers (MDFHS/A/145). Participants’ rights were protected throughout the study. They were informed of their freedom to withdraw at any time without any penalty, thereby upholding the ethical principles of autonomy and self-determination. All interviews with participants who provided written consent were conducted in a private room, thus ensuring the principle of privacy. Participants were assured that their personal details and interview data would remain confidential. Data were stored in a password-protected computer to enhance confidentiality. Informed consent and participant authorisation were obtained prior to data collection.

## Qualitative findings

### Participants’ profile

The socio-demographics of the eight participants are shown in Table 1. Eight soldiers were interviewed until data saturation was reached. Five soldiers were aged between 22 and 25 years, while three were aged between 30 and 35 years. Two soldiers were married, and six were not married. Seven were Christians, and one was a Muslim. In terms of education, seven soldiers had the Malawi School Certificate of Education, while one had a Bachelor’s degree in Environmental Health. Regarding ethnicity, one participant was Ngoni, while seven were Chewa.

**TABLE 1:** Participants’ profile.

Participant	Age range (Years)	Marital status	Religion	Education	Tribe
P1	22–25	Single	Christian	MSCE	Chewa
P2	22–25	Single	Christian	MSCE	Chewa
P3	22–25	Single	Christian	MSCE	Chewa
P4	22–25	Married	Muslim	BSc in Environmental Health	Ngoni
P5	30–35	Single	Christian	MSCE	Chewa
P6	30–35	Single	Christian	MSCE	Chewa
P7	30–35	Married	Christian	MSCE	Chewa
P8	22–25	Single	Christian	MSCE	Chewa

### Presentation of themes and sub-themes on psychological experiences

In this section, the researcher began the write-up of the report (Braun & Clarke 2020) in a concise, coherent, logical and non-repetitive manner, quoting the verbatim which best described the identified themes. Three major themes and six sub-themes emerged from the study. The three major themes were immediate, short-term and long-term psychological experiences (See Table 2). These are described as follows:

**TABLE 2:** Themes and sub-themes.

Themes	Sub-themes
1. Immediate psychological experiences	1.1. Immediate emotional response 1.2. Perceived physical discomfort
2. Short-term psychological experiences	2.1. Adaptation to physical restrictions 2.2. Feelings of self-consciousness
3. Long-term psychological experiences	3.1. Shifted self-perceptions 3.2. Prolonged physical and emotional effects

#### Theme 1: Immediate psychological experiences

The immediate experiences reported by participants revolved around initial emotional and physical reactions, including feelings of vulnerability, pain and concerns about recovery. These were coded into two main sub-themes: immediate emotional response and perceived physical discomfort. These are described as follows:

**Sub-theme 1.1: Immediate emotional response:** Soldiers reported feeling a range of emotions immediately following the procedure, such as anxiety, fear and relief. Many expressed that, although they felt uncertain about potential complications, they were also relieved that the procedure was over.

One participant shared:

‘I was worried about what would happen after, but I also felt like I had accomplished something important. I was worried that the wound will keep bleeding and cause excessive pain after the operation.’ (P1, 22–25, Single, Christian)

‘There was fear before the procedure, but once it was done, I felt lighter, like a burden had been lifted. I was afraid that my sexual performance would be affected and wondered how long it would take to perform again and resume normal duties at work.’ (P5, 30–35, Single, Christian)

**Sub-theme 1.2: Perceived physical discomfort:** Pain and discomfort were frequently cited as immediate physical reactions post-circumcision. Participants described pain in the circumcised area that, for some, led to difficulty performing daily tasks and a sense of vulnerability. As one participant described:

‘I could feel pain every movement; even walking was uncomfortable. It made me feel weak and vulnerable. I was experiencing that something is not right in my body with general body aches, the desire to see the swollen part and not having enough energy to walk around.’ (P2, 22–25, Single, Christian)

‘The discomfort was constant, and it made me worry if this pain would have long-term effects. The discomfort was constant especially during urination and when I want to walk around to stretch.’ (P6, 30–35, Single, Christian)

Another participant mentioned the psychological toll of the pain, stating that it caused them to worry about recovery and long-term implications. The emotional distress and discomfort often created a mixture of relief and anxiety, with some expressing concerns about whether the surgeons had performed the procedure properly and if it would have lasting impacts on their health and functioning.

#### Theme 2: Short-term psychological experiences

Participants commonly reported psychological adjustments as they adapted to their healing bodies. These adjustments were classified into two main sub-themes: adaptation to physical restrictions and feelings of self-consciousness. Feelings of self-consciousness sometimes resulted in isolation, as participants felt that sharing their experiences might expose them to judgment or discomfort from others. These are described as follows:

**Sub-the me 2.1: Adaptation to physical restrictions:** Many participants noted that the physical limitations imposed by the healing process required significant mental and emotional adjustment. Soldiers reported a temporary decrease in physical performance, affecting their ability to participate fully in military activities and other duties. For some, this led to frustration and feelings of inadequacy, particularly as they compared their current physical state to pre-circumcision performance. One participant reflected:

‘Not being able to train as usual made me feel like I wasn’t fully myself; I felt restrained and a bit helpless. I am a soldier, and we have a routine of exercises; therefore, after this, it was difficult to follow that routine.’ (P3, 22–25, Single, Christian)

‘The limitations affected my confidence. I felt like I couldn’t perform my duties properly.’ (P8, 22–25, Single, Christian)

**Sub-theme 2.2: Feelings of self-consciousness:** Participants also experienced self-consciousness during the initial recovery period, particularly regarding the visibility of their healing process. Some soldiers reported feeling embarrassed or hesitant to discuss their condition with peers, leading to social withdrawal. For instance, one soldier mentioned:

‘I felt everyone could tell that something was different. It was hard to talk about, so I avoided it. In a way, you cannot talk freely about something that will protect you from infections acquired during our routine work-related duties, like spending one week in a jungle without bathing.’ (P6, 30–35, Single, Christian)

Another stated:

‘It is not easy to get circumcised when you are an adult, so you become self-conscious of body image changes, because culturally, in our context of Malawi, it is not an operation you are obliged to do.’ (P7, 30–35, Married, Christian)

#### Theme 3: Long-term psychological experiences

Participants provided insight into their evolving perceptions, lasting physical effects, and the influence of VMMC on their sense of self. The long-term experiences were grouped into two key themes: shifted self-perception and prolonged physical and emotional effects. For some, the experience was transformative, influencing their self-image and attitudes towards personal health and hygiene.

**Sub-theme 3.1: Shifted self-percept ion:** Over time, many participants reported a shift in how they perceived themselves and their bodies post-circumcision. Some felt an increased sense of cleanliness and pride, associating VMMC with positive health benefits. One participant stated:

‘It has changed how I see myself. I feel more confident about my health. This operation has changed me in the way I think and feel about myself – physically, socially, and emotionally. I no longer care what other people feel about me.’ (P4, 22–25, Married, Muslim)

Another reflected:

‘I feel like it’s healthier. I don’t regret it, because now I know I’m always clean, even if I get involved in unhealthy routine duties. I am not worried anymore about infections like HIV and other sexually transmitted infections.’ (P7, 30–35, Married, Christian)

**Sub-theme 3.2: Prolonged physical and emotional effects:** A few participants reported prolonged physical and emotional effects even months after the procedure. These included greater sexual satisfaction and ongoing self-consciousness about intimate relationships and cleanliness. For some, these effects continued to shape their interactions and relationships, particularly in personal hygiene and intimate settings. As expressed by participants:

‘I still feel different. Not in pain, but it feels like increased sexual enjoyment with my wife, and this has enhanced our relationship.’ (P4, 22–25, Married, Muslim)

‘The long-term benefits outweigh the challenges. I feel more in control of my hygiene and have no fear of any potential negative effects.’ (P1, 22–25, Single, Christian)

## Discussion

The data suggest that VMMC has a distinct psychological impact on soldiers, beginning with an intense immediate response that includes feelings of vulnerability, anxiety and discomfort. In the short term, the analysis indicates a period of physical and psychological adjustment, marked by frustration over physical limitations and self-consciousness regarding healing. Over the long term, the findings reveal a complex process of identity shift and self-perception adjustment, where some soldiers associate circumcision with positive health perceptions, while others continue to experience concerns related to personal hygiene and sexual satisfaction, which in turn enhances relationships. These findings directly address the research questions and provide insights into the evolving nature of soldiers’ psychological experiences post-circumcision.

### Socio-demographic characteristics

The demographic characteristics of the participants provide essential context for understanding the psychological experiences of soldiers who underwent VMMC. This diverse age distribution highlights a mix of early-career and mid-career military personnel, which may influence their psychological responses to VMMC differently because of varying life experiences and responsibilities, as age appears to play a significant role in shaping perceptions and motivations. Patel et al. (2018) conducted a study across South Africa, Tanzania and Zimbabwe, finding that younger adolescents (aged 10–14) were more likely to seek VMMC because of external advice, such as suggestions from parents or school officials.

In contrast, older adolescents (aged 15–19) were more motivated by personal health benefits, including protection against HIV and other sexually transmitted infections. In addition, younger adolescents exhibited less awareness of the partial protection VMMC offers against HIV compared to their older counterparts (Patel et al. 2018). These findings suggest that age-related differences in motivations and understanding of VMMC could influence the psychological responses of individuals undergoing the procedure. Younger individuals may require more targeted education and counselling to address their specific concerns and informational needs, while older individuals might benefit from discussions that reinforce their health-related motivations. This multifaceted approach could help address the diverse psychological experiences associated with VMMC across different age groups.

Marital status could play a role in shaping psychological experiences, particularly in how participants perceive the effect of VMMC on intimate relationships. Married participants may focus more on the procedure’s implications for their spouses, while unmarried participants might view it from an individual health or social perspective. The predominance of Christianity as a religious belief may influence participants’ attitudes towards VMMC, especially in how they interpret its cultural, spiritual or health-related significance. The variation in participants’ educational backgrounds suggests differing levels of health literacy, which could impact their understanding of and psychological adjustment to the procedure. The tribal identities of Chewa and Ngoni may also have cultural implications for participants’ perceptions of VMMC. Circumcision practices and attitudes towards the procedure can vary across ethnic groups in Malawi, and this was consistent with the findings of Mphepo et al. (2023).

### Immediate psychological experiences

The findings from this study not only reflect several psychological experiences observed in prior research but also highlight aspects unique to the military context of the sample. The immediate psychological responses, including anxiety, pain and vulnerability, are consistent with Uberoi et al. (2021) and Yang et al. (2014), who noted that individuals often experience a heightened sense of anxiety and discomfort before and after VMMC. The high anxiety levels observed in this study align with Yang et al.’s (2014) findings, which indicate that psychological factors can amplify pain perception. Yang et al. (2014) noted that anxiety could amplify the perception of pain, suggesting that the soldiers’ emotional responses may have influenced their physical experiences. Given soldiers’ rigorous physical training and social expectations of resilience, they may experience amplified anxiety and stress before circumcision, as they are accustomed to a physically demanding and high-stakes environment. Moreover, Josephine, Grace and Kabunga (2023) indicated that anxiety was a reason for the low uptake of VMMC, thus suggesting the need for adequate psychological preparation, including pre-procedure counselling and emotional support to address specific military-related anxiety triggers.

Participants reported heightened emotional reactions following the circumcision, including fear and relief. These reactions are primarily linked to uncertainties about the procedure’s success and concerns about potential complications, such as infection or prolonged pain. Soldiers often expressed heightened sensitivity to these risks because of their need to maintain physical performance and fulfil demanding military roles. These findings align with Uberoi et al. (2021) and Yang et al. (2014), who observed similar heightened levels of anxiety and emotional distress before and immediately after VMMC. This is particularly relevant in a military context, where high-stakes environments and expectations of resilience may exacerbate emotional responses. Furthermore, the sense of relief expressed by participants after completing the procedure suggests a psychological release from the anticipation of pain or complications. This dual experience of anxiety and relief highlights the complex emotional landscape soldiers navigate during the immediate recovery period.

Participants consistently reported pain and discomfort as significant physical reactions immediately after the procedure. This physical discomfort often led to feelings of vulnerability and a perceived loss of control, as soldiers were temporarily unable to perform their routine tasks or engage in physical activities critical to their roles. These experiences are consistent with the findings of Mehmetoğlu (2023), who reported that physical discomfort is a common immediate reaction to circumcision, often accompanied by emotional distress. Soldiers’ heightened sense of vulnerability is notable, as it contrasts with their usual perception of physical strength and endurance, which is integral to their identity and roles. This perceived vulnerability could be further amplified in military settings, where soldiers are expected to maintain physical readiness at all times. Bossio, Pukall and Steele (2017) suggested that the psychological impact of temporary physical limitations may be more pronounced in individuals with high physical demands, such as soldiers, compared to civilians. The combination of emotional and physical challenges during this period underscores the need for comprehensive pre-procedure and post-procedure counselling. Such counselling could help soldiers anticipate and manage both emotional responses (e.g. anxiety) and physical reactions (e.g. pain), thereby promoting a smoother recovery process.

### Short-term psychological experiences

Short-term psychological experiences also highlighted concerns about body image and masculinity, echoing findings from Agha (2024) and Bossio et al. (2017). These studies observed that men, particularly soldiers, may struggle with altered self-perceptions and concerns about physical appearance following circumcision, which could impact their confidence and social interactions. Soldiers in this study similarly reported feelings of self-consciousness and social withdrawal, aligning with the observations of Agha (2024). Notably, the social dynamics in a military setting, where physical performance and appearance are highly valued, may exacerbate concerns around body image and masculinity, underscoring the value of incorporating body image counselling into post-circumcision support.

In the short term, many soldiers experienced frustration and emotional distress as a result of the physical limitations imposed by the healing process. These restrictions affected their ability to fully participate in military training and duties, leading to feelings of inadequacy and a temporary loss of their physical identity as soldiers. Participants described this adjustment as mentally taxing, as they struggled to reconcile their usual physical capabilities with their temporary limitations. These findings align with Agha (2024) and Szivak and Kraemer (2015), who noted that men in military contexts, where physical readiness is not only a requirement but also a measure of identity and pride, are likely to experience intensified challenges. Soldiers accustomed to rigorous training routines may experience greater psychological strain when faced with temporary physical restrictions (Tornero-Aguilera et al. 2024). Additionally, the frustration expressed by participants highlights the need for clear communication about the expected healing timeline and reassurance about their ability to regain full functionality. Thus, short-term counselling sessions focusing on coping strategies during recovery could address this frustration and provide emotional support.

Feelings of self-consciousness were another prominent theme in the short-term psychological experiences of soldiers. Participants reported heightened awareness of their healing process, with some expressing embarrassment or fear of judgment from their peers. This self-consciousness often led to social withdrawal and avoidance of discussions about their condition, as soldiers sought to shield themselves from potential stigma or discomfort. These findings echo Bossio et al. (2017) and Ngalande et al. (2006), who observed that concerns about body image and masculinity are prevalent in men recovering from circumcision. In a military environment, where physical strength and appearance are closely tied to conceptions of masculinity, these feelings of self-consciousness can be particularly significant. The fear of judgment reported by participants may stem from the perception that discussing personal health matters, especially those related to circumcision, could be seen as a sign of weakness. This aligns with previous research indicating that social dynamics in male-dominated environments often discourage open discussions about vulnerability or health concerns. To mitigate these experiences, it is essential to incorporate body image counselling and peer support into post-circumcision care programmes. Normalising discussions about the recovery process and creating a safe environment for soldiers to share their experiences can reduce feelings of isolation and foster a sense of camaraderie.

### Long-term psychological experiences

In the long term, participants in this study experienced a composite of positive self-perceptions and enduring discomfort, which differs from the findings of Aydoğdu, Azizoğlu & Okur. (2022) and Aydogmus et al. (2016). Aydoğdu et al. (2022) observed that some soldiers experienced enduring changes in body image and self-esteem, and Aydogmus et al. (2016) found that long-term psychological adjustment was generally positive when adequate social support was provided. Soldiers who perceived the procedure positively often cited improved health and cleanliness, similar to the positive long-term outcomes noted by Kigozi et al. (2008) regarding sexual satisfaction and body confidence. For others, however, ongoing sensitivity and self-consciousness indicate that not all soldiers experience a complete psychological adjustment, highlighting the need for extended post-procedure psychological and social support to ensure long-term well-being.

Over time, many participants reported a positive shift in their self-perception, often associating VMMC with improved health, cleanliness and personal hygiene. These changes contributed to an increased sense of pride and self-confidence, as participants viewed the procedure as a proactive step towards better overall health. These findings align with Kigozi et al. (2008), who found that men often associate circumcision with increased cleanliness, sexual satisfaction and improved body confidence. For soldiers, the association between circumcision and personal health is particularly meaningful, as their roles often demand peak physical performance and well-being. The transformative effect of VMMC on self-perception suggests that, for many, the procedure serves as a milestone in personal development, fostering a more positive relationship with their bodies. However, this shift is not universal, as some participants reported lingering challenges related to self-image and confidence.

While many participants experienced positive changes, a subset reported enduring physical and emotional effects that continued to shape their experiences months after the procedure. These effects included ongoing sensitivity, heightened self-consciousness in intimate settings, and concerns about the procedure’s long-term implications. These findings diverge slightly from the observations of Aydogmus et al. (2016). While Aydogmus et al. (2016) found that most men reported positive psychological adjustment when adequate social support was provided, the participants in this study highlighted enduring emotional challenges, suggesting the need for more sustained post-procedure support. The military context may amplify these experiences, as soldiers must navigate unique pressures related to physical readiness, intimate relationships and social dynamics within their units. For those who reported enhanced sexual satisfaction and relationship improvement, the procedure was seen as a contributor to personal and relational well-being, consistent with findings from Kigozi et al. (2008). However, for others, the ongoing self-consciousness underscores the variability in long-term psychological adjustment.

### Limitations of the study

There was a chance of information bias, as participants might have provided inaccurate information. This was mitigated by probing with additional questions. Recall bias was possible, as participants were asked to remember their circumcision experience. To mitigate recall bias, only participants circumcised within the past 2 years were selected. Researcher bias was also expected, since the researchers had prior knowledge of the concepts under study. However, the researchers employed reflexivity and engaged a diverse research team to maintain transparency during data collection and analysis.

### Recommendations

Immediate and short-term psychological support should include pre-procedure counselling to address anxiety, pain management and recovery expectations. Educational workshops are also recommended to prepare soldiers for the psychological and physical experiences associated with VMMC. Moreover, future studies should investigate the long-term psychological and physical effects of VMMC on larger military populations to better understand how soldiers’ experiences evolve. Lastly, policies should be developed to allow soldiers to perform lighter duties or non-physical tasks during the recovery period.

## Conclusion

This study explored the psychological experiences of soldiers who underwent VMMC at the Engineers Battalion in Kasungu, Malawi, focusing on immediate, short-term and long-term effects. The findings revealed that soldiers initially experienced anxiety, pain and vulnerability, followed by frustration over physical limitations and self-consciousness in the short term. Over time, many reported positive self-perceptions linked to improved hygiene and health benefits, while others continued to experience lingering concerns about body image and masculinity. These results highlight the need for tailored psychological support, including pre-procedure and post-procedure counselling, peer support systems, and policies that accommodate recovery needs. Despite limitations such as recall and researcher bias, this study provides critical insights into the psychological impact of VMMC in a military setting, offering valuable guidance for future research, policy development and mental health interventions.
